# Prevalence of Respiratory Viruses in Children With Acute Respiratory Infections in Shanghai, China, From 2013 to 2022

**DOI:** 10.1111/irv.13310

**Published:** 2024-05-09

**Authors:** Li Zhang, Yuanping Wang, Hongmei Xu, Lipeng Hao, Bing Zhao, Chuchu Ye, Weiping Zhu

**Affiliations:** ^1^ Shanghai Pudong New Area Center for Disease Control and Prevention Shanghai China

**Keywords:** child, prevalence, respiratory tract infections, respiratory viruses

## Abstract

**Background:**

A variety of viruses can cause acute respiratory infections (ARIs), resulting in a high disease burden worldwide. To explore the dominant viruses and their prevalence characteristics in children with ARIs, comprehensive surveillance was carried out in the Pudong New Area of Shanghai.

**Methods:**

Between January 2013 and December 2022, the basic and clinical information, and respiratory tract specimens of 0–14 years old children with ARIs were collected in five sentinel hospitals in Shanghai Pudong. Each specimen was tested for eight respiratory viruses, and the positive rates of different age groups, case types (inpatient or outpatient) were analyzed.

**Results:**

In our study, 30.67% (1294/4219) children with ARIs were positive for at least one virus. Influenza virus (IFV) was the most commonly detected respiratory virus (349/4219, 8.27%), followed by respiratory syncytial virus (RSV) (217/4219, 5.14%), para‐influenza virus (PIV) (215/4219, 5.10%), and human coronavirus (HCoV, including 229E, OC43, NL63, and HKU1) (184/4219, 4.36%). IFV was the leading respiratory virus in outpatients aged 5–14 years (201/1673, 12.01%); RSV was the most prevalent respiratory virus in both inpatients (61/238, 25.63%) and outpatients (4/50, 8.00%) for ARI patients aged <6 months old. For PIV, HMPV, HCoV, and HRV, the risk of infection usually was higher among young children. Co‐infection with more than two viruses was seen in 3.25% (137/4219).

**Conclusions:**

IFV and RSV played important roles in ARIs among children, but the risk populations were different. There are needs for targeted diagnosis and treatment and necessary immunization and non‐pharmaceutical interventions.

## 1 Introduction

Acute respiratory infections (ARIs) are an important cause of morbidity and mortality among children, and lower respiratory infections were the second leading cause of DALYs (disability‐adjusted life‐years) for children younger than 10 years in 2019 [1]. Viruses such as influenza virus (IFV), respiratory syncytial virus (RSV), human metapneumovirus (HMPV), para‐influenza virus (PIV), adenovirus (ADV), human coronavirus (HCoV), human rhinovirus (HRV), and human bocavirus (HBoV) are considered to be the common pathogens causing ARIs [2, 3, 4, 5, 6]. Symptoms of influenza infections in children are usually mild, but morbidity usually exceeds that in adults [3]. In the case of RSV, there were 33.0 million RSV‐associated acute lower respiratory infection episodes in children aged 0–60 months in 2019 globally [2]. HMPV, PIV, ADV, HCoV, HRV, and HBoV have been detected in children with ARIs in many studies [6, 7, 8, 9, 10, 11]. Although most respiratory viral infections are mild, they may cause life‐threatening illness such as pneumonia, particularly in high‐risk groups such as neonates [7]. ARIs in children also impose a substantial economic burden; for example, a mean annual cost of €87.1 million has been reported for Spanish children associated with ARIs due to RSV [12].

Children with ARIs often have similar clinical presentations, and no individual clinical feature allows any particular type of viral infection to be ruled in or ruled out [13]. Therefore, laboratory testing is necessary to accurately diagnose respiratory infections and can help to prescribe specific antiviral drugs for certain viruses, such as oseltamivir for influenza [14]. However, it is difficult to carry out laboratory testing for every ARI case due to limited medical resources and large population. Thus, long‐term sentinel etiological surveillance of children with ARIs is necessary to understand the local ARI virus spectrum its changing trend, so as to improve the targeted clinical diagnosis and treatment, and provide a basis for appropriate infection prevention and control measures.

A comprehensive surveillance network covering outpatient and inpatient cases of acute respiratory infections has been implemented in the Pudong New Area of Shanghai, Eastern China, since 2013. Although a study about viral pathogens among elderly people with acute respiratory infections has been published based on this surveillance [15], no studies have been conducted in children. Indeed, despite research analyzing the epidemiology of respiratory pathogens in children with lower respiratory tract infections or severe acute respiratory tract infections in Shanghai [16, 17], they only involved single‐center studies based on information from one hospital. Our present study aimed to detect respiratory viruses in children with ARIs via multicenter surveillance, to explore the dominant viruses and their prevalence characteristics in children in this region, so as to provide reference for disease prevention policy making and clinical practice.

## 2 Materials and Methods

### 2.1 Ethical Approval

This study was based on the data obtained from the Comprehensive Surveillance of Acute Respiratory Infections in the Pudong New Area, which was implemented by the Shanghai Pudong New Area Center for Disease Control and Prevention. The data analysis and study protocols of the project were reviewed and approved by the Medical Ethics Committee of Shanghai Pudong New Area Center for Disease Control and Prevention.

### 2.2 Study Design

Between January 2013 and December 2022, active surveillance of pediatric patients aged 0–14 years old with ARIs was conducted in the Pudong New Area of Shanghai. To truly reflect the prevalence of respiratory viruses in children with ARIs in Pudong, five hospitals were selected as sentinel sites based on their location, catchment area, and patient volume [18], including four general hospitals and one pediatric hospital (Table 1). All participating hospitals carried out patient enrolment and specimen collection according to the monitoring implementation plan formulated by Shanghai Pudong New Area Center for Disease Control and Prevention (Pudong CDC). Before and during the surveillance was carried out, all healthcare workers participating in surveillance work in the sentinel hospitals had accepted training to be qualified for recruiting patients and sample collection.

**TABLE 1:** List of sentinel hospitals for children ARI surveillance in Shanghai Pudong New Area, China.

Sentinel hospitals	Monitoring departments			
	Pediatric outpatient department	Pediatric inpatient unit	Pediatric infection ward	Pediatric intensive care unit
Shanghai Children's Medical Center			√^a^	√
Shanghai East Hospital	√			
Shanghai Pudong Hospital	√	√		
Shanghai Yangsi Hospital	√			
Shanghai Pudong New District Zhoupu Hospital		√		

^a^ “√” indicates that ARI surveillance in children was carried out.

### 2.3 Patient Enrolment and Specimen Collection

ARIs were defined as follows: (1) acute onset within 10 days; (2) at least one of the following symptoms/signs: sore throat, cough, expectoration, nasal congestion, runny nose, chest pain, tachypnea, and abnormal pulmonary breath sounds; and (3) with or without fever. Patients with confirmed diagnoses of noninfectious respiratory diseases, such as asthma and respiratory tumors, were excluded.

For each week, approximately 1–5 patients with ARIs aged 0–14 years old in each of the sentinel hospitals were enrolled in this study via convenience sampling. In 2013 and 2022, the sample size was small due to the pilot phase of surveillance and the impact of COVID‐19, respectively.

The attending physician selected enrolled patients who met the ARI definition based on their self‐reported symptoms during the visit. For each enrolled patient, the basic and clinical information were recorded in a questionnaire formulated by Pudong CDC, and the throat swab or sputum were collected and stored in 2 mL of viral transport media (VTM, Yocon, Beijing, China), which was transported at 2°C–8°C within 24 h to Pudong CDC for laboratory testing. Specimens that could not be transported immediately were stored temporarily at −20°C. The parents/guardians of the participants in this study were required to provide brief verbal consent during patient enrolment, which was recorded in each questionnaire by the physician.

### 2.4 Laboratory Method

Each specimen was tested within 24 h of collection by polymerase chain reaction (PCR) for eight viral pathogens: IFV (including subtypes A, B, and C), HRV, PIV (including subtypes 1–4), ADV, RSV (including subtypes A and B), HMPV, HCoV (including 229E, OC43, NL63, and HKU1), and HBoV. Real‐time reverse transcription polymerase chain reaction (RT–PCR) was performed using a LightCycler480II Real‐Time Fluorescent PCR Instrument (Roche, Switzerland) and a RespiFinder SMART 22 FAST Kit (RespiFinder, Lot: PF2500‐SF, Netherlands). If not examined within 48 h, the collected specimens were stored at −70°C until tested.

### 2.5 Data Management and Statistical Analysis

After the questionnaires were collected from the sentinel hospitals by Pudong CDC, several professionals were designated to entered the questionnaire data and the corresponding test results into the standard informatics system established by the Chinese Centers for Disease Control and Prevention. Statistical analysis was performed using R 4.2.3 (R Core Team, R: A language and environment for statistical computing. R Foundation for Statistical Computing, Vienna, Austria). Descriptive statistics included frequency analysis for categorical variables and medians and IQRs for continuous variables. The chi‐square test or Fisher's exact test was used for categorical variables. Wilcoxon rank‐sum or Kruskal–Wallis tests were employed for continuous variables, as appropriate. Joinpoint regression model was used to analyze age trends, which was performed using Joinpoint regression Program (V 5.0); *p* values < 0.05 were considered statistically significant.

## 3 Results

### 3.1 Study Population

A total of 4219 children with ARIs were included in this study. There were 2281 males and 1938 females. Their median age was 4 years old (interquartile ranges [IQR] 2–7 years), among which 11.73% were under 1 year old, 16.59% were 1–2 years old, 22.68% were 3–4 years old, and 48.99% were 5–14 years old. A total of 2844 were outpatients, and 1375 were inpatients. There was no significant difference in sex distribution between the outpatient and inpatient cases (*p* = 0.164), though the median age of the inpatients (3, [IQR] 0–5) was younger than that of the outpatients (5, [IQR] 3–8). Outpatient specimens were continuously collected from 2013 to 2022, with an average of 6.1 specimens per week. Inpatient specimens were continuously collected from 2016 to 2022, with an average of 4.4 specimens per week.

### 3.2 Overall Virus Detection

Of the 4219 specimens obtained from children with ARIs, 1294 (30.67%) were positive for at least one virus (Table 2). A significantly higher rate of any virus positivity was found for the inpatients than the outpatients (36.80% vs. 27.71%, *p* < 0.001). There were no significant differences between sexes in either outpatients (27.24% in males vs. 28.24% in females, *p* = 0.583) or inpatients (39.08% in males vs. 33.93% in females, *p* = 0.056). Among the outpatients, any virus positive rates were comparable between different age groups (*p* = 0.547). While among the inpatients, any virus positive rates differed significantly between age groups (24.51% in < 6 months old, 13.44% in 6–11 months old, 42.37% in 1–2 years old, 31.53% in 3–4 years old, 21.57% in 5–14 years old, *p* < 0.001). The annual rate of positivity among outpatient cases varied from 5.88% (2021) to 50.78% (2017); that of inpatient cases varied from 10.64% (2021) to 64.80% (2016).

**TABLE 2:** Characteristics and virus test results for children with ARIs.

Characteristics	Total			Outpatient			Inpatient		
	All	Positive for any virus	Positive rate (%)	All	Positive for any virus	Positive rate (%)	All	Positive for any virus	Positive rate (%)
Sex									
Female	1938	582	30.03	1328	375	28.24	610	207	33.93
Male	2281	712	31.21	1516	413	27.24	765	299	39.08
Age group									
< 6 months	288	138	47.92	50	14	1.78	238	124	24.51
6–11 months	207	87	45.45	80	19	2.41	127	68	13.44
1–2 years old	700	246	35.14	379	110	29.02	321	136	42.37
3–4 years old	957	290	30.30	662	197	29.76	295	93	31.53
5–14 years old	2067	533	25.79	1673	448	26.78	394	85	21.57
Sample year									
2013	188	46	24.47	188	46	24.47	0	\	\
2014	222	60	27.03	222	60	27.03	0	\	\
2015	225	88	39.11	225	88	39.11	0	\	\
2016	398	225	56.53	202	98	48.51	196	127	64.80
2017	362	205	56.63	193	98	50.78	169	107	63.31
2018	758	261	34.43	594	187	31.48	164	74	45.12
2019	846	272	32.15	475	133	28.00	371	139	37.47
2020	537	72	13.41	423	57	13.48	114	15	13.16
2021	537	45	8.38	255	15	5.88	282	30	10.64
2022	146	20	13.70	67	6	8.96	79	14	17.72
Total	4219	1294	30.67	2844	788	27.71	1375	506	36.80

### 3.3 Virus Spectrum

**FIGURE 1:**
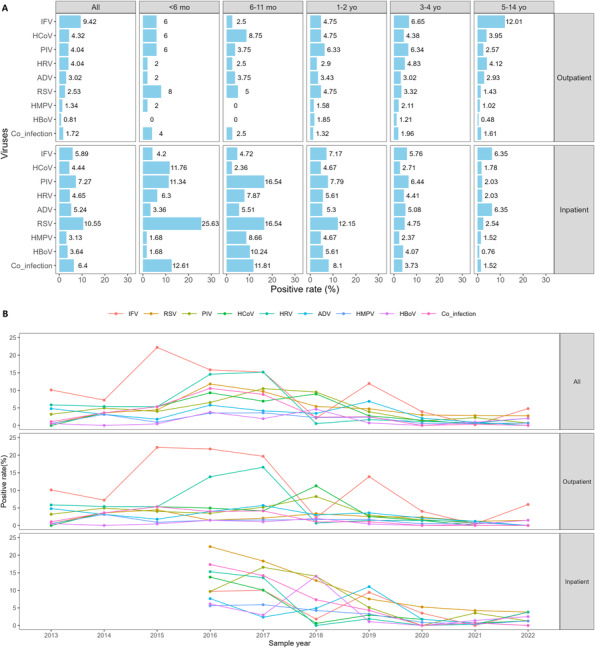
Positive rates of respiratory viruses in children with ARIs among outpatients and inpatients. (A) Based on age group. (B) Based on sample year. Mo, months old; Yo, years old.

IFV was the most commonly detected respiratory virus in the 4219 specimens (349, 8.27%), followed by RSV (217, 5.14%), PIV (215, 5.10%), HCoV (184, 4.36%), HRV (179, 4.24%), ADV (158, 3.74%), HMPV (81, 1.92%), and HBoV (73, 1.73%). Further case type‐specific analysis revealed that IFV (268/2844, 9.42%) was the most common virus among outpatients, followed by HCoV (123/2844, 4.32%), PIV (115/2844, 4.04%), and HRV (115/2844, 4.04%) (Figure 1A). For inpatients, the most common viruses were RSV (145/1375, 10.55%), PIV (100/1375, 7.27%), IFV (81/1375, 5.89%), and ADV (72/1375, 5.24%).

For ARI patients aged < 6 months old, RSV was the most prevalent respiratory virus in both inpatients (61/238, 25.63%) and outpatients (4/50, 8.00%) (Figure 1A). RSV and PIV were the top two ranked viruses both in inpatients aged 6–11 months old and 1–2 years old. Nevertheless, for ARI outpatients aged 6–11 months old, HCoV was the most prevalent respiratory virus (7/80, 8.75%) and RSV ranked second (4/80, 5.00%); for ARI outpatients aged 1–2 years old, the detection rates of IFV and HCoV and RSV were second only to PIV. For ARI patients aged 3–4 years old, the top two viruses in outpatients were IFV and PIV, which were the same in inpatients, but the ranking was reversed. For ARI patients aged 5–14 years old, IFV became the most common respiratory virus in outpatients (201/1673, 12.01%), and the positive rates of ADV and IFV were the same and the highest (25/394, 6.35%) in inpatients.

Each virus had higher detection rates from 2016 to 2019 but lower detection rates from 2020 to 2022 (Figure 1B). Two annual peaks (2015, 2019) and three annual troughs (2014, 2018, and 2021) were alternately observed for the positive rates of IFV from 2013 to 2022. From each year's test results, IFV was the most frequently detected respiratory virus in outpatients every year except for 2018 and 2021. RSV was the most frequently detected respiratory virus in inpatients in 2016, 2017, 2020, and 2021. The ranking of positive rates of other respiratory viruses often changed from year to year. HCoV was the most frequently detected virus in outpatients only in 2018. ADV was the most frequently detected virus in inpatients only in 2019.

### 3.4 Age‐Specific Virus Detection

**FIGURE 2:**
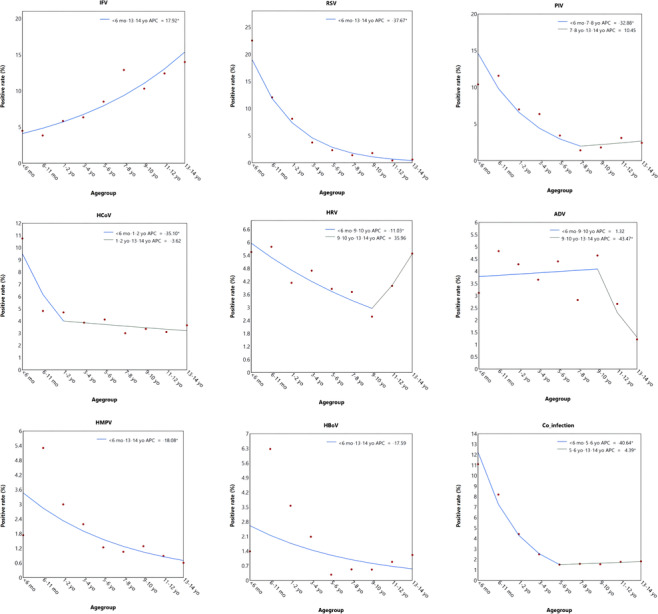
The Joinpoint regression of the positive rates of each tested virus in children with ARIs based on age group. A red point indicates the mean positive rate of patients in terms of age, and the colored curves indicate fitted Joinpoint regression patterns by the red points. Legends give the annual percent change (APC) value of each fitted curve for each tested virus. Asterisk (*) indicates that the APC is significantly different from zero at two‐sided *p* < 0.05. The *p* values were not adjusted for multiple comparisons.

Among the detected viruses, IFV showed a different age‐specific detection pattern from other viruses (Figure 2). IFV was observed with the lowest positive rates in infant patients aged < 1 year old, followed by a persistent increase to the highest level in children aged 13–14 years old (significant APC > 0). RSV and HMPV showed persistent decrease trends, with higher rates observed in infant patients and the lowest rates observed in children aged 13–14 years old (significant APC < 0). PIV, HCoV and HRV were observed with higher positive rates in younger children patients, followed by a significant downward trend before certain age. The positive rate of ADV decreased from 9–10 years old, with the lowest positive rate in children patients aged 13–14 years old. No statistically significant age‐specific change in positive rate of HBoV was observed.

### 3.5 Mixed Infection of Viruses

Among the 4219 children with ARIs, co‐infection with more than two viruses was seen in 3.25% (137/4219). The detection rate of co‐infection was 1.72% (49/2844) in outpatients and 6.40% (88/1375) in inpatients respectively, with a significant difference (*p* < 0.001) (Figure 1A). The detection rate of co‐infection showed a downward trend from infants aged < 6 months old to children aged 5–6 years old, and then showed a upward trend (significant APC > 0) (Figure 2).

Types of co‐infection were complex (Table 3). A total of 2.77% (117/4219) of the pediatric ARI patients were infected with 2 viruses, and 0.47% (20/4219) were infected with ≥ 3 viruses. The most commonly observed co‐infection was observed among HCoV and another virus, with 45 samples (accounting for 32.85% of all co‐infection samples), followed by IFV co‐infection with another virus (23/137, 16.79%) and RSV co‐infection with another virus (17/137, 12.41%). Specifically, HCoV + PIV co‐infection was most frequent, with 15 samples (accounting for 10.95% of all co‐infection samples), followed by HCoV + RSV co‐infection (11/137, 8.03%), and IFV + RSV co‐infection (8/137, 5.84%).

**TABLE 3:** Common coinfection types of respiratory viruses in children with ARIs. Only common coinfection types are listed.

Coinfection types	Number	Percentage (%)	Detection rate (%) (*N* = 4219)
Two viruses	117	85.40	2.77
HCoV + 1 virus	45	32.85	1.07
HCoV + PIV	15	10.95	0.36
HCoV + RSV	11	8.03	0.26
Other	19	13.87	0.45
IFV + 1 virus	23	16.79	0.55
IFV + RSV	8	5.84	0.19
IFV + HRV	7	5.11	0.17
Other	8	5.84	0.19
RSV + 1 virus	17	12.41	0.40
RSV + PIV	7	5.11	0.17
RSV + HBoV	6	4.38	0.14
Other	4	2.92	0.09
Other two viruses	32	23.36	0.76
HRV + HMPV	7	5.11	0.17
PIV + HMPV	7	5.11	0.17
Other	18	13.14	0.43
Three viruses	20	14.60	0.47
PIV + HCoV + HRV	3	2.19	0.07
IFV + RSV + HRV	2	1.46	0.05
Other	15	10.95	0.36
Total	137	100.00	3.25

## 4 Discussion

In this study, we retrospectively analyzed detection of eight respiratory viruses (IFV, RSV, PIV, HCoV, HRV, ADV, HMPV, and HBoV) in 4219 children with ARIs over a span of 10 years in Shanghai, China. Our results provide a distinctive profile of prevalence of respiratory viruses in children with ARIs.

Among children with ARIs enrolled in our study, 30.67% were positive for at least one virus, which is close to the rates of respiratory viruses in the literature (24.5%–46.9%) [6, 11, 19]. Several community surveillance studies have reported respiratory virus positivity rates ranging from approximately 55.5% to 76.8% among children with ARIs [7, 8, 9].

Of the viruses examined, IFV, RSV, PIV, HCoV, HRV, and ADV were frequently identified in both inpatients and outpatients. By comparing the virus spectrum between different age groups in inpatients and outpatients, we found that IFV was the most common virus detected in outpatients (in 9.42% of outpatients), and also the leading virus in the 5‐ to 14‐year‐old group. IFV mainly causes an upper respiratory infection, and only a small number of cases lead to serious illness requiring hospitalization, but the direct costs and indirect costs associated with treating an infection cannot be ignored [3, 20]. In particular, school‐age children infected with influenza may have reduced sick leave due to milder symptoms, which often results in transmission of influenza in highly crowded school settings. Nevertheless, RSV was found to be the most common virus detected in inpatients (in 10.55% of inpatients), and was identified as the leading virus in outpatients and inpatients aged < 6‐month‐old, and inpatients aged 6‐ to 11‐month‐old and 1‐ to 2‐year‐old. RSV was likely to be related to hospitalization in children, consistent with other studies [8, 21]. A systematic analysis conducted in 2019 also showed that RSV contributes substantially to the global morbidity and mortality burden in children aged 0–60 months, especially during the first 6 months of life and in low‐income and middle‐income countries [2]. In the 3‐ to 4‐year‐old group, higher detection rates were found for both IFV and PIV, and the virus spectra were similar for inpatients and outpatients.

Since January 2020, Shanghai has continuously reported COVID‐19 cases and experienced the first wave of COVID‐19 from March to May 2022 [22, 23]. In response, Shanghai took prevention and control measures such as restricting airplane travel, reducing gatherings, and wearing masks [24]. As shown in this study and others, respiratory virus infections decreased in 2020, 2021 and 2022, which might be associated with unprecedented changes in societal and human behaviors; nevertheless, IFV and RSV still played important roles in ARIs [25]. In our study, IFV showed a rebound upward trend and became the dominant respiratory virus in 2022 after an extremely low detection rate in 2021; RSV remained the dominant respiratory virus of children hospitalized with ARIs in 2020 and 2021. Likewise, the CDC reported a relative increase in influenza virus activity during the 2022–2023 influenza season in the United States (compared with the 2021–2022 season), with a peak value of more than 25% of samples positive for IFV in December 2022 [26]. In 2021, early epidemics of RSV infection were evidenced in several countries including the United States and Japan, with virus detection rates exceeding the seasonal peak of pre–COVID‐19 years [27].

Trends in viral positive rates with age can help in identifying groups of children at the highest risk of infection. Our study showed that IFV positive rates increased with age, with higher risk of infection in school‐aged children, consistent with other literatures [3, 6]. In contrast, the positive rates of RSV and HMPV decreased with age, with the highest risk of infection in infants. Analogously, a systematic review and meta‐analysis published in 2023 revealed that RSV‐related disease incidence decreased with increasing age among children ≤ 5 years and that the incidence in the 0‐ to 1‐year‐old group was three times higher than that in the 2‐ to 5‐year‐old group [28]. For PIV, HCoV, and HRV, the risk of infection usually was higher among young children, and decreased with age, which might be related to the continuous improvement of children's immunity; however, the reduction in risk was no longer evident after a certain age and might even increase, possibly related to the increased range of activities in elder children and the higher prevalence of these viruses in adults [5, 15, 29].

In our study, 3.25% of children with ARIs were co‐infected with more than two respiratory viruses, which is lower than the range of 9.3% to 36.2% reported in previous studies [6, 7]. However, we found that the detection rate rose to 9.90% in children under 1 year old, revealing that young children are more likely to have high rates of co‐infection. Our findings showed that HCoV, IFV, and RSV were the respiratory viruses most frequently involved in co‐infections. Nonetheless, the clinical significance of co‐infection is not fully understood, with prior studies reporting both greater or the same severity of clinical outcomes with respiratory viral co‐infections [30].

Several limitations exist in this study. First, the number of specimens collected was lower at the beginning of the surveillance and during the COVID‐19 epidemic. However, our preliminary results clearly reveal the predominant viral pathogen in children with ARIs, providing primary background information. Second, due to the limitations in testing capabilities during COVID‐19, samples collected in our study were not tested for COVID‐19. Regardless, due to prevention and control measures, no large‐scale epidemics of COVID‐19 occurred in the community population in Shanghai from 2020 to 2021 [22, 24], during which COVID‐19 was not the main pathogen of ARIs in community population. Third, our study did not include bacterial pathogens, which are also important pathogens causing acute respiratory infections. However, several factors, such as the usage of antibiotics prior to treatment, might lead to low detection rates of respiratory bacterial pathogens, making it difficult to obtain valuable results.

Despite these limitations, the results of our study may be helpful in proposing targeted prevention control measures. Influenza vaccines are the best available intervention to reduce the public health impact of influenza epidemics. Meanwhile, given that influenza viruses are detected more often in school‐age children, non‐pharmaceutical interventions (NPIs) such as hand hygiene, face masks, isolation of infected persons, school closures might be effect measures to reduce transmission in school and community [3]. In response to the highest risk of RSV infection in infants, passive immunization programs for infants may reduce the burden of RSV disease [2]. Clinicians can also carry out targeted diagnosis and treatment according to the age distribution characteristics of viral infections.

## Author Contributions

**Li Zhang:** conceptualization, data curation, formal analysis, methodology, writing–original draft, writing–review and editing. **Yuanping Wang:** conceptualization, data curation, methodology, project administration, writing–review and editing. **Hongmei Xu:** conceptualization, data curation, methodology, project administration, writing–review and editing. **Lipeng Hao:** methodology, resources, supervision. **Bing Zhao:** methodology. **Chuchu Ye:** conceptualization, methodology, project administration, writing–review and editing. **Weiping Zhu:** conceptualization, methodology, project administration, writing–review and editing.

## Conflicts of Interest

The authors declare no conflicts of interest.

### Peer Review

The peer review history for this article is available at https://www.webofscience.com/api/gateway/wos/peer‐review/10.1111/irv.13310.

## Data Availability

The data that support the findings of this study are available from the corresponding author, Chuchu Ye, upon reasonable request.
